# Metabolism studies of paeoniflorin in rat liver microsomes by ultra-performance liquid chromatography coupled with hybrid quadrupole time-of-flight mass spectrometry (UPLC-Q-TOF-MS/MS)

**DOI:** 10.1098/rsos.180759

**Published:** 2018-10-03

**Authors:** L. J. Zhu, S. S. Sun, Y. X. Hu, Y. F. Liu

**Affiliations:** 1 School of Pharmaceutical Sciences, Liaoning University, Shenyang 110036, People's Republic of China; 2 Natural Products Pharmaceutical Engineering Technology Research Center of Liaoning Province, Shenyang 110036, People's Republic of China

**Keywords:** paeoniflorin, rat liver microsomes, metabolites, UPLC-Q-TOF-MS/MS

## Abstract

To explore metabolism mechanism of paeoniflorin in the liver and further understand intact metabolism process of paeoniflorin, a rapid, convenient and effective assay is described using ultra-performance liquid chromatography coupled with hybrid quadrupole time-of-flight mass spectrometry (UPLC-Q-TOF-MS/MS). The strategy was confirmed in the following primary processes: firstly, different concentration of paeoniflorin, rat liver microsomes, coenzymes and different incubated conditions were optimized to build a biotransformation model of rat liver microsomes *in vitro* by high performance liquid chromatography with diode array detection (HPLC-DAD); secondly, the metabolites of paeoniflorin in rat liver microsomes were detected and screened using UPLC-Q-TOF-MS/MS by comparing the total ion chromatogram (TIC) of the experimental group with those of control groups; finally, the molecular formulae and corresponding chemical structures of paeoniflorin metabolites were identified by comparing the MS and MS/MS spectra with the self-constructed database and simulation software. Based on this analytical strategy, 20 metabolites of paeoniflorin were found and 6 metabolites (including four new compounds) were tentatively identified. It was shown that hydrolysis and oxidation were the major metabolic pathways of paeoniflorin in rat liver microsomes, and the main metabolic sites were the structures of pinane and the ester bond. These findings were significant for a better understanding of the metabolism of paeoniflorin in rat liver microsomes and the proposed metabolic pathways of paeoniflorin might provide fundamental support for the further research in the pharmacological mechanism of Paeoniae Radix Rubra (PRR).

## 1. Introduction

Paeoniflorin, a bioactive monoterpene glucoside in the root of Paeoniae Radix Rubra (PRR), has numerous pharmacological effects such as antioxidant [1], anti-inflammatory [2], analgesic [3] and muscle relaxant [4]. Recently, paeoniflorin has gained extensive attention due to its various pharmacological actions and fewer toxic side effects. Meanwhile, some studies on the metabolism of paeoniflorin *in vivo* have been carried out [5–7]. Based on the scholar Takeda's research [6,8], a comparative study between germ-free rats and conventional rats after paeoniflorin administration indicated that paeoniflorin could hardly be metabolized in the gut wall, liver or lung, and its transformation in the intestine led to an extremely low bioavailability. Consequently, few studies on metabolites of paeoniflorin *in vitro* especially in liver microsomes have been reported.

According to the scholar Hattori's and our researches [9,10], paeoniflorin could be hydrolysed, oxidized and reduced by human intestinal microflora (HIM). Obviously, the liver is regarded as the essential site for drug biotransformation on account of the large number and high abundance of drug-metabolizing enzymes especially cytochrome P450 (CYP450) which could catalyse some reactions like hydrolysis, oxidation and conjunction [11,12]. Therefore, we proposed that paeoniflorin might have similar metabolic reactions in the liver. In order to elaborate the integrated metabolic process and further explore the metabolic mechanism of paeoniflorin, it is necessary to investigate the biotransformation of paeoniflorin in the liver. The metabolic model consisting of liver microsomes and coenzymes is applied for the metabolic study *in vitro* due to its powerful capability [13–15] with the same temperature and physiological environment *in vivo* [16]. The method is feasible to study the prototype drugs and their metabolites even in a low concentration because of the easily controlled experimental conditions. The interference of endogenous substances can be eliminated and the metabolites can be detected accurately. Therefore, the metabolic model of liver microsomes was applied in this study by its excellent imitating characteristics *in vivo*, good reproducibility, and convenience in operation.

High-resolution mass spectrometry (HRMS) such as hybrid quadrupole time-of-flight mass spectrometry (Q-TOF) analyser is applied to identify the metabolites because of its high-throughput and sensitive analytical technique [17,18]. It has demonstrated the great advantages for the structure analysis of components in herbal extracts and their metabolites with simple construction, low time consumption, wide mass range, high sensitivity and resolution, and low consumption of samples [19–21]. In our strategy, an optimal incubation system was formed by optimizing metabolic conditions and then metabolites of paeoniflorin were detected and identified using UPLC-Q-TOF-MS/MS. The metabolic study of paeoniflorin in liver microsomes could provide a further understanding of the metabolism of paeoniflorin and it might be significant for the study of the pharmacological mechanism of PRR.

## 2. Experimental

### 2.1. Chemicals and reagents

**Figure 1.**
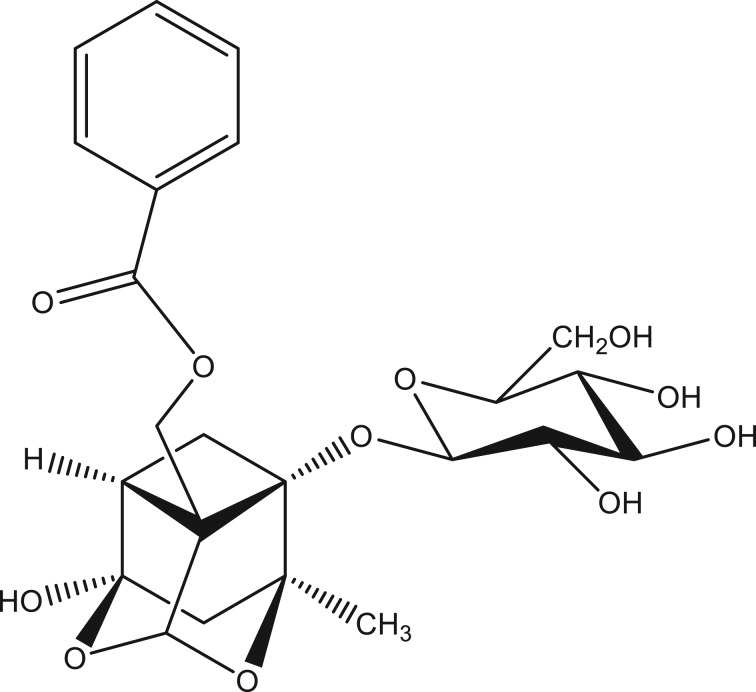
The chemical structure of paeoniflorin.

Paeoniflorin was purchased from the National Institute for the Control of Pharmaceutical and Biological Products (Beijing, China; catalogue number: 110736) and its chemical structure is shown in figure 1. Tris (hydroxymethyl) aminomethane was purchased from Sinopharm Chemical Reagent Co., Ltd (Shanghai, China; catalogue number: 301883392). Bovine Serum Albumin (BSA) was purchased from Shanghai Yuanye Biological Technology Co., Ltd (Shanghai, China; catalogue number: S12012). Phenobarbital sodium injection was purchased from the Fourth People's Hospital of Shenyang (Shenyang, China). Glucose-6-phosphate (G-6-P) disodium salt and glucose-6-phosphate dehydrogenase (G-6-P DH) were purchased from Nanjing Duly Biotech Co., Ltd (Nanjing, China; catalogue numbers: C0020 and E0050, respectively). β-nicotinamide adenine dinucleotide phosphate (NADP) disodium salt and β-nicotinamide adenine dinucleotide reduced (NADH) disodium salt were purchased from Shanghai Regal Biology Technology Co., Ltd (Shanghai, China; catalogue numbers: 24292-60-2 and 606-68-8, respectively). Acetonitrile and methanol were of HPLC grade and purchased from Shandong Yuwang Industrial Co., Ltd Chemical Branch (Dezhou, China). Purified water was used for all analysis. All other chemicals were the highest purity commercially available.

### 2.2. Instrumentation and condition

#### 2.2.1. HPLC-DAD analysis conditions

Experiments for optimizing metabolic conditions were performed on an Agilent 1200 HPLC system (Agilent, USA), which consisted of a quaternary pump, an online degasser, an autosampler, a column oven and a diode array detector. The separation was achieved by a Dikma Diamonsil™ ODS C_18_ column (5 µm, 250 × 4.6 mm i.d.). The mobile phase comprised acetonitrile and water (30 : 70). The flow rate was 1 ml min^−1^, and the detection was performed at a wavelength of 230 nm under the operating temperature of 30°C. The injection volume was 10 µl.

#### 2.2.2. UPLC-MS/MS analysis conditions

The detection of metabolites of paeoniflorin in rat liver microsomes was conducted on Dikma Diamonsil™ ODS C_18_ column (1.8 µm, 100 × 2.1 mm i.d.) using a Waters UPLC system (Waters, USA). The flow rate was 0.2 ml min^−1^, and the column was operated at 30°C. The injection volume was 2 µl. A gradient elution of A (an aqueous solution of 1% formic acid, *v/v*) and B (acetonitrile and 1% formic acid, *v/v*) was applied. The gradient elution was as follows: started at 90% A and 10% B, then to 70% A and 30% B at 2 min, 40% A and 60% B at 4 min, and kept with 5% A and 95% B from 8 min to 11 min. After that, the gradient elution was restored to the initial condition for 4 min.

For mass detection, Waters LCT Premier XE Q-TOF system with a Lock-Spray™ source was carried out in a negative-ion mode (ESI^−^) across the mass range of *m*/*z* 100–1000 Da. The set parameters were applied for MS detection: capillary voltage, 2.8 kV; desolvation temperature, 300°C; source temperature, 110°C; desolvation gas flow, 500 l h^−1^. The MS/MS detection conditions were as follows: capillary voltage, 2.8 kV; desolvation temperature, 300°C; sample cone voltage, 35.0 V; extraction cone voltage, 3.0 V; source temperature, 110°C; cone gas flow, 50 l h^−1^; desolvation gas flow, 500 l h^−1^; collision energy, 15–35 eV. Mass during acquisition was calibrated by applying an external reference (Lock-Spray™) composed of a 0.2 ng ml^−1^ solution of leucine enkephalin. Through a lockspray interface, the leucine enkephalin was injected at a flow rate of 0.02 ml min^−1^, which occurred with a reference ion at 554.2620 [M − H]^−^. The scan time of lockspray was 0.5 s with the time span of 15 s. Data were set on average of three scans.

### 2.3. Preparation of rat liver microsomes

Sprague-Dawley male rats (SPF degree, 240 ± 20 g, certificate no. SCXK2015-0001), purchased from Liaoning Changsheng Biotechnology Co., Ltd (Benxi City, Liaoning Province, China), were acclimated for 7 days on a 12 h light–dark cycle at 20–26°C. To induce cytochrome P450 (CYP450) enzymes, rats were given intraperitoneally phenobarbital sodium in physiological saline solution at a dose of 60 mg kg^−1^ once per day for 3 days. Before the experiments, the rats were fed with water at random and fasted overnight. The rats were killed 24 h after the last dose by pulling their cervical vertebra and their livers were quickly removed and then washed several times with ice-cold 1.15% KCl solution immediately to remove the residual blood. Liver microsomes were obtained by the method of Burstein & Kupfer [22]. The liver was weighed after it was dried by filter paper and cut into pieces and homogenized at 4000 r.p.m. in an ice-cold bath. The resulting microsomal pellet was then suspended in 10 mM potassium phosphate buffer (pH 7.4) and centrifuged at 10 000*g* for 20 min at 4°C. The supernatant fraction was centrifuged again at 105 000*g* for 70 min at 4°C. Then the supernatant was discarded and the liver microsomes were suspended uniformly into Tris-HCl buffer (pH 7.4), the volume of which was about one-quarter of quondam supernatant, and was stored at −80°C until use.

The protein concentration of the prepared rat liver microsomes was determined by Lowry protein assay employing BSA as the standard protein [23]. The standard protein BSA was taken and diluted to standard protein solutions at 0, 64.0, 128.0, 192.0, 256.0 and 320.0 µg ml^−1^ with water. The standard protein solution was monitored by ultraviolet spectrophotometry at 660 nm. Each concentration was executed in triplicate and the calibration curve was plotted according to the measured results. Then the rat liver microsomes were diluted by 100 times water and monitored by ultraviolet spectrophotometry at the same conditions. The protein concentration of the prepared rat liver microsomes was obtained by the calibration curve of BSA.

### 2.4. Optimization of microsomal incubation conditions and preparation of the sample

According to the references [15,24], eight groups (including four experimental groups and four control groups) were set to investigate metabolic conditions in the NADPH-generating system.

Each experimental group was conducted in parallel three times. As shown in table 1, the rat liver microsomes and different concentrations of NADP, NADH, G-6-P, G-6-P DH and MgCl_2_ were quantitatively dissolved with 50 mmol l^−1^ Tris-HCl buffer. The mixture was pre-incubated in a shaking water bath at 37°C for 5 min. Then the paeoniflorin solution (dissolved in pH 7.4 Tris-HCl buffer) was quantitatively added into the mixture and the reaction was triggered. The final total volume of the microsomal incubation system was 1 ml. Oxygen was circulated on the surface of the incubation mixture every 20 min. After 120 min, the reaction was terminated by adding triple methanol immediately. The mixture was shaken for 30 s and centrifuged at 5000 r.p.m. for 10 min. The supernatant was carefully transferred to an EP tube and dried under nitrogen. The residue was then dissolved again by 1 ml methanol and the solution was shaken for 30 s and centrifuged at 5000 r.p.m. for 10 min again. The supernatant was filtered through a 0.22 µm microfiltration membrane for HPLC analysis. Four control groups were set to investigate the role of the NADPH-generating system for metabolic incubation. Control group 1 was composed of NADP, NADH, G-6-P, G-6-P DH, Mg^2+^, paeoniflorin and inactivated rat liver microsomes in Tris-HCl buffer. To inactivate the metabolic system, the liver microsomes and coenzymes were added into Tris-HCl buffer and then kept in boiling water bath for 10 min. For control group 2, only the liver microsomes were added. Control group 3 included only paeoniflorin. Control group 4 consisted of liver microsomes and paeoniflorin, but no coenzymes were added.

**Table 1.** Optimization of the NADPH-generating system conditions. Eight groups were set to optimize incubation conditions and evaluate the metabolic effect. Compared experimental group 3 with control groups 1–4, the characteristic peaks were screened by total ion chromatograms.

groups	exp. 1	exp. 2	exp. 3	exp. 4	ctrl. 1	ctrl. 2	ctrl. 3	ctrl. 4
liver microsomes (mg ml^−1^)	2.0	2.0	2.0	2.0	2.0 (inactivated)	2.0	—^a^	2.0
NADP (mmol l^−1^)	1.0	1.0	1.0	0.5	1.0	—	—	—
NADH (mmol l^−1^)	—	1.0	0.5	0.5	0.5	—	—	—
G-6-P (mmol l^−1^)	10.0	10.0	10.0	10.0	10.0	—	—	—
G-6-P DH (IU ml^−1^)	1.0	1.0	1.0	1.0	1.0	—	—	—
Mg^2+^ (mmol l^−1^)	4.0	4.0	4.0	4.0	4.0	—	—	—
paeoniflorin (mg ml^−1^)	0.1	0.1	0.1	0.1	0.1	—	0.1	0.1
conversion rate (%)	0.2	3.5	28.6	7.2	3.6	n.d.^b^	0	0
RSD (%) *n* = 3	1.6	1.5	4.4	2.2	2.8	n.d.^b^	0.2	2.7

^a^Not added.

^b^Not detected.

Next, the remaining content of paeoniflorin was detected by HPLC-DAD analysis and determined by the calibration curve of paeoniflorin. The acquisition of the calibration curve of paeoniflorin was described as follows: Paeoniflorin standard solutions were detected by HPLC-DAD at 0.02, 0.04, 0.06, 0.08, 0.09, 0.10 and 0.11 mg ml^−1^. The linear regression equation resulted from the least square method in which the peak areas were taken as the *y*-axis and the corresponding concentrations were taken as the *x*-axis. Then the optimal metabolic condition of paeoniflorin in rat liver microsomes was confirmed by comparing the calculated conversion rates within different groups. In order to detect and identify the metabolites of paeoniflorin in rat liver microsomes, the group which had the highest conversion rate, together with control group 2 and 3, was selected to the UPLC-MS/MS analysis.

## 3. Results and discussion

### 3.1. Determination of protein content

**Figure 2.**
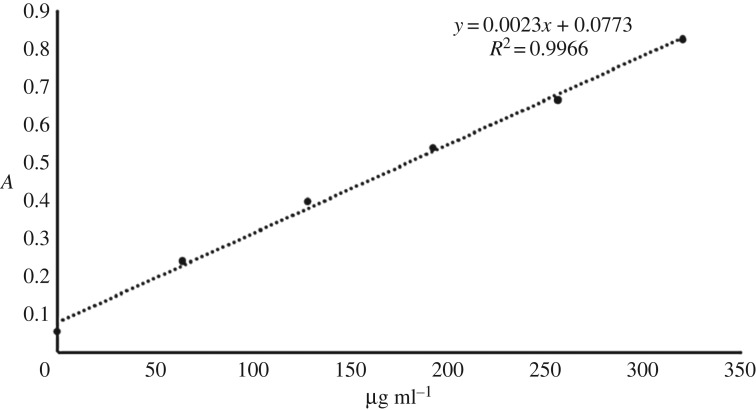
The calibration curve of the protein solution.

The absorbance of the BSA standard protein solution was determined with ultraviolet spectrophotometry. The least square method was applied to the linear regression and the linear relation of the standard protein was very good at 0–320.0 µg ml^−1^. The regression equation was *y* = 0.0023*x* + 0.073 (*R*^2^ = 0.9966). The rat liver microsomes were diluted by 100 times water, 0.5 ml of which was measured and the absorbance was 0.673. According to the calibration curve of the standard protein solution, the protein concentration of rat liver microsomes was 25.88 mg ml^−1^ and the protein calibration curve of the BSA standard is shown in figure 2.

### 3.2. Determination of paeoniflorin content

**Figure 3.**
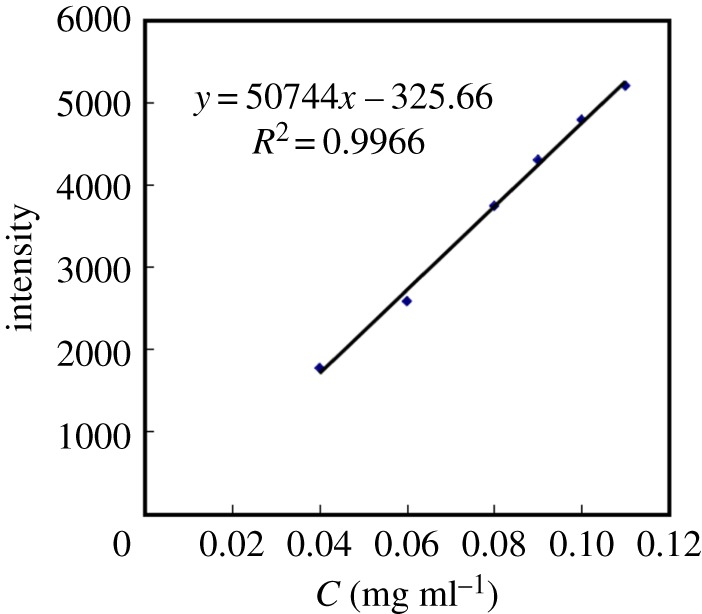
The calibration curve of paeoniflorin.

The absorbance of the paeoniflorin standard solution was determined with HPLC-DAD. The linear regression was achieved by the least square method and the linear relation of the paeoniflorin standard solution was perfect at 0.02–0.11 mg ml^−1^. The regression equation was *y* = 54 318*x* − 256.55 (*R*^2^ = 0.9966) and the calibration curve of paeoniflorin is shown in figure 3.

### 3.3. Microsomal incubation

**Figure 4.**
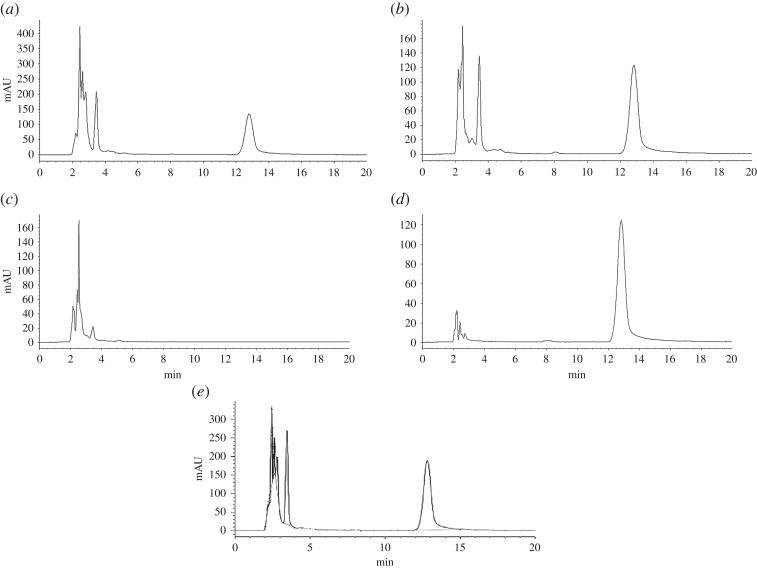
The HPLC-DAD chromatograms of the samples of one experimental group and four control groups, where the chromatographic peak at 12.80 min is paeoniflorin. (*a*) Experimental group 3; (*b*) control group 1; (*c*) control group 2; (*d*) control group 3; (*e*) control group 4.

Eight groups (four experimental groups and four control groups) were set to evaluate metabolic effect in the NADPH-generating system. The conversion rate of each group was the proportion of the difference between the initial and remaining content of paeoniflorin divided by the initial content of paeoniflorin added. The results of chromatograms for experimental group 3 and four control groups are shown in figure 4, in which the chromatographic peak at 12.80 min is paeoniflorin. The conversion rates of different groups are presented in table 1. These results indicate that paeoniflorin was metabolized in the model of rat liver microsomes. Experimental group 3, which had the highest conversion rate, was the optimal metabolic group. As a result, the optimal incubation system was ascertained including 2.0 mg ml^−1^ rat liver microsomes, 1.0 mmol l^−1^ NADP, 0.5 mmol l^−1^ NADH, 10.0 mmol l^−1^ G-6-P, 1.0 IU ml^−1^ G-6-P DH, 4.0 mmol l^−1^ Mg^2+^ and 0.1 mg ml^−1^ paeoniflorin.

### 3.4. Metabolites identification

In this study, metabolites of paeoniflorin were identified by software and database established by our research group including InChIKey, CAS, molecular formula, accurate molecular weight and fragment ions and references of 264 chemical compounds. Firstly, the newly appeared chromatographic peaks were distinguished by MS-DIAL V1.57 software to compare total ion chromatogram (TIC) of the experimental group with those of control groups 2 and 3. Next, the MS/MS data of discovered metabolites were obtained by setting corresponding retention time periods of those newly appeared chromatographic peaks in the range of 15–35 eV collision energy using UPLC-Q-TOF-MS/MS. Finally, the molecular formulae and chemical structures of metabolites were identified and confirmed by MS2 Analyzer V2.1 software, references, and CFM-ID (http://cfmid.wishartlab.com/). In our strategy, 20 metabolites of paeoniflorin were found and six metabolites were identified preliminarily (including four new metabolites) and the identified metabolites were demonstrated as follows.

M1 was eluted at 1.65 min, displayed quasi-molecular ion [M − H]^−^ at 267.0672 and the formula was assigned as C_16_H_12_O_4_. A series of distinctive fragment ions were shown at 135, 133 and 79 in the MS/MS spectrum of M1. The characteristic ion 135 (C_8_H_7_O_2_) was formed when the structure of pinane was lost from the quasi-molecular ion [M − H]^−^. And the product ion at 133 was deduced as the fragment of the structure of pinane which was lost from the precursor ion. The diagnostic fragment ion 79 was detected and considered to be the fragment of a benzene ring. Therefore, oxidization and demethylation probably occurred on the structure of pinane. Based on the analysis of MS/MS data and the result of software simulation, M1 was tentatively identified as oxidation demethyldeglucopaeoniflorin.

Based on the MS/MS data, the molecular formula of M2 was considered to be C_18_H_20_O_6_. It showed fragment ions at 313, 227, 135, 121 and 79. In its MS/MS spectrum, the fragment ion *m/z* 313 was observed, which was formed by the loss of 18 Da (elemental composition: H_2_O) from the quasi-molecular ion [M − H]^−^ at 331.1172. The fragment ion 227 was formed by the loss of the benzoyl group (104 Da) from [M − H]^−^. The product ions at 135 and 121 were observed and inferred to be the characteristic ions of the benzoxy group. Thus, according to the previous study [25] and the result of software simulation, M2 was heuristically assigned as deglucomethylpaeoniflorin.

Metabolite M3 exhibited the quasi-molecular ion at 333.1336 [M − H]^−^ and the elemental formula was assigned as C_18_H_22_O_6_. It was eluted at 7.07 min. The diagnostic products ions were at 315, 275, 257, 211, 199 and 135. The fragment ions 315 and 257 were formed by the loss of H_2_O (18 Da) from the quasi-molecular ion [M − H]^−^ and the fragment ion 275, respectively. The fragment ion 275 was obtained by successive elimination of CO_2_ (40 Da) from the fragment ion 315. The fragment ions 211 and 199 were respectively assigned as C_11_H_15_O_4_ and C_10_H_15_O_4_, which were obtained by the loss of the benzoxy group from the quasi-molecular ion [M − H]^−^. On the basis of the result of software simulation, M3 was provisionally deduced as deglucomethylalbiflorin.

M4 was eluted at 7.33 min and the quasi-molecular ion was observed at 315.1246 [M − H]^−^ (C_18_H_20_O_5_). The distinctive fragment ions at 257, 121 and 79 were observed in its MS/MS spectrum. The fragment ion 257 was formed by loss of C_2_H_2_O_2_ (58 Da) from the quasi-molecular ion [M − H]^−^. And the characteristic ions 121 and 79 were considered to be the benzoxy group and the benzene ring, respectively. According to the result of software simulation, M4 was tentatively deduced as oxidation deglucomethylalbiflorin.

M5 was eluted at 7.62 min and the quasi-molecular ion peak of 343.1385 [M − H]^−^ appeared, which was assigned as C_16_H_24_O_8_. M5 showed characteristic ions at 325, 313, 181, 163, 151 and 109. The fragment ion 325 was assigned as C_16_H_21_O_7_ and was formed by the loss of H_2_O (18 Da) from the quasi-molecular ion [M − H]^−^. In a previous study [26], the fragment ions at 181, 163, 151 and 109 were reported. Thus, M5 was provisionally identified as mudanpioside F.

Metabolite M6, 299.0938 [M − H]^−^ (C_17_H_16_O_5_), was appeared at 8.94 min. In the MS/MS spectrum, the fragment ions at 283, 267, 121 and 77 were observed. The characteristic product ion at 283 was formed by the loss of CH_4_ (16 Da) from the quasi-molecular ion [M − H]^−^. When an O (16 Da) was lost from the fragment ion 283, the fragment ion 267 was formed. The fragment ions 121 and 77 were assigned as C_7_H_5_O_2_ and C_6_H_5_ to be the diagnostic fragment of the benzoxy group and the benzene ring, respectively. Therefore according to the result of software simulation, M6 was heuristically identified as oxidation paeoniflorin.

### 3.5. Metabolic pathways of paeoniflorin

**Figure 5.**
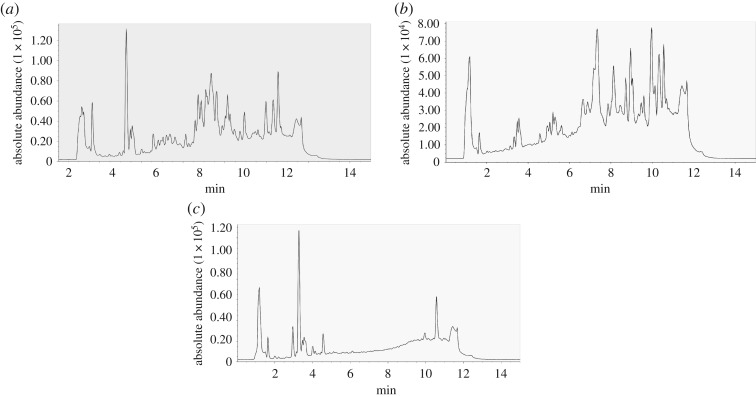
Total ion chromatograms of the samples of one experimental and two control groups. (*a*) Experimental group 3; (*b*) control group 2; (*c*) control group 3.

**Figure 6.**
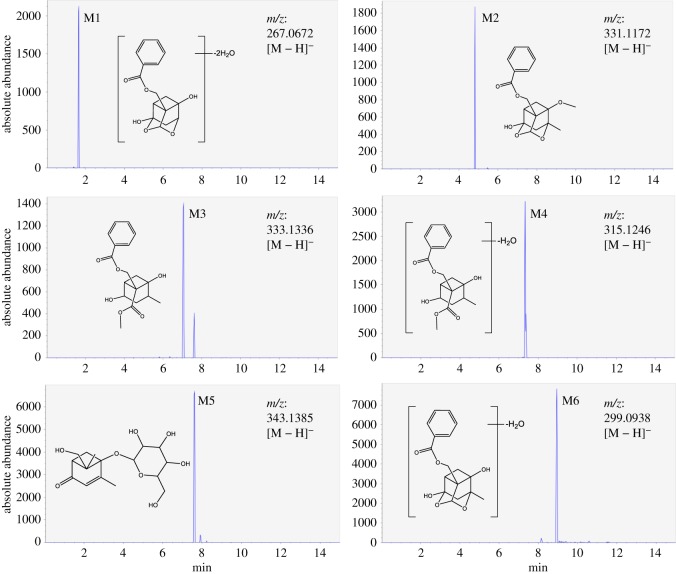
Extracted ion chromatograms of the identified metabolites of paeoniflorin in rat liver microsomes.

**Figure 7.**
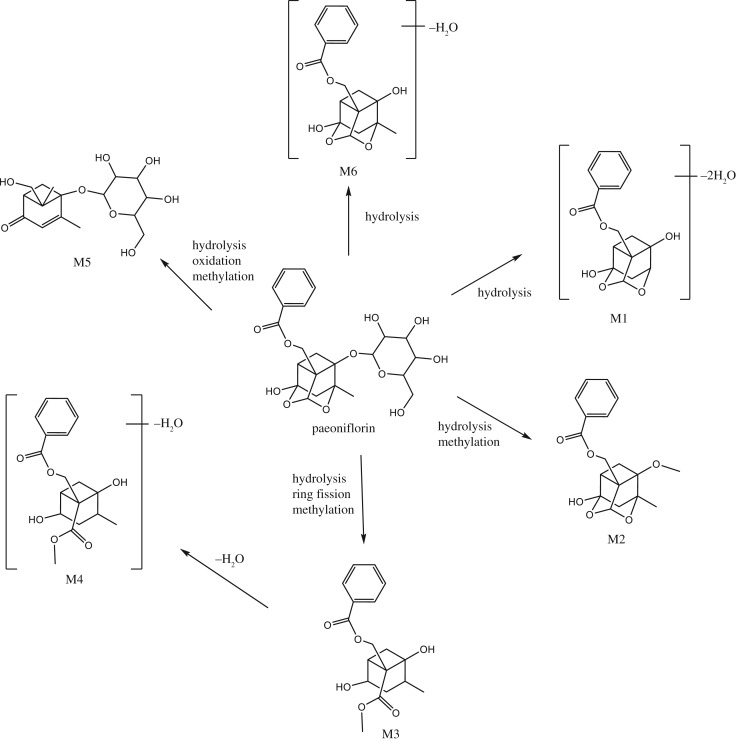
Proposed major metabolic pathway of paeoniflorin in rat liver microsomes.

This study explored the metabolism of paeoniflorin *in vitro* by rat liver microsomes. Twenty metabolites of paeoniflorin were found, in which six metabolites of paeoniflorin (including four new metabolites) were identified. The total ion chromatograms of experimental group 3 and control groups and the extracted ion chromatograms of identified metabolites are presented in figures 5 and 6, respectively. The quasi-molecular ions, retention times, molecular formulae and fragment ions of those metabolites are summarized in table 2 and their proposed major metabolic pathways of paeoniflorin in rat liver microsomes are shown in figure 7. The result demonstrated that the major metabolic reactions were oxidation, hydrolysis and methylation and the main metabolic sites were the structure of pinane and the ester bond. In addition, hydrolysis on the site of glucosidic bond was the primary metabolic pathway, which was in accordance with the scholar Hattori's research [9]. However, some metabolites of paeoniflorin were not hydrolysed at the ester linkage in rat liver microsomes. The steric hindrance might obstruct the catalysis of hydrolase at the ester linkage due to the structure of paeoniflorin.

**Table 2.** MS and MS/MS spectrum data of the metabolites of paeoniflorin in rat liver microsomes including retention times (Rt), molecular formula, measured *m/z*, predicted *m/z*, the error between measured *m/z* and predicted *m/z*, and the corresponding major fragment ions and names of identified metabolites.

peak no.	Rt (min)	molecular formula	meas. (*m/z*)	pred. (*m/z*)	error (ppm)	major fragment ions (*m/z*)	identification result
1 (M1)	1.65	C_16_H_12_O_4_	267.0672 [M − H]^−^	267.0663	3.37	135, 133, 79	oxidation demethyldeglucopaeoniflorin
2	1.64	C_14_H_24_O_9_	335.1380 [M − H]^−^	335.1347	9.85		unidentified
3	3.33	C_27_H_34_O_13_	611.1967 [M + FA − H]^−^	611.1981	−2.29		unidentified
4 (M2)	4.81	C_18_H_20_O_6_	331.1172 [M − H]^−^	331.1187	−4.53	313, 227, 135, 121, 79	Deglucomethylpaeoniflorin
5	6.14	—^a^	287.2871	—	—	—	unidentified
6	6.71	—	314.3142	—	—	—	unidentified
7	6.87	—	337.3150	—	—	—	unidentified
8	6.96	—	269.2683	—	—	—	unidentified
9 (M3)	7.07	C_18_H_22_O_6_	333.1336 [M − H]^−^	333.1344	−2.10	315, 275, 257, 211, 199, 135	Deglucomethylalbiflorin
10	7.09	C_15_H_34_N_4_O	285.2683 [M − H]^−^	285.2660	8.06	—	unidentified
11	7.27	C_22_H_42_O_2_	337.3121 [M − H]^−^	337.3112	2.71	—	oxidation C_22_H_42_O
12 (M4)	7.33	C_18_H_20_O_5_	315.1246 [M − H]^−^	315.1238	2.54	257, 121, 79	oxidation deglucomethylalbiflorin
13	7.46	—	295.2953	—	—	—	unidentified
14	7.58	—	271.2883 [M − H]^−^	—	—	—	unidentified
15 (M5)	7.62	C_16_H_24_O_8_	343.1385 [M − H]^−^	343.1398	−3.79	325, 313, 181, 163, 151, 109	mudanpioside F
16	7.63	C_20_H_40_O_2_	311.2925 [M − H]^−^	311.2955	−9.64	—	unidentified
17	8.13	C_12_H_14_N_2_O_7_	297.0742 [M − H]^−^	297.0728	4.71	—	unidentified
18	8.24	C_22_H_40_O	319.3006 [M − H]^−^	319.3006	0.00	—	unidentified
19	8.34	C_22_H_42_O	321.3145 [M − H]^−^	321.3163	−5.51	—	reduction C_22_H_40_O
20 (M6)	8.94	C_17_H_16_O_5_	299.0938 [M − H]^−^	299.0925	4.35	283, 267, 121, 77	oxidation paeoniflorin

^a^Unknown.

## 4. Conclusion

In this work, a rapid, convenient and effective strategy was carried out. In this strategy, ultra-performance liquid chromatography coupled with hybrid quadrupole time-of-flight mass spectrometry (UPLC-Q-TOF-MS/MS) was used to detect metabolites of paeoniflorin in rat liver microsomes. The optimal transformation system was ascertained by calculating the conversion rates after incubation. For metabolite identification, UPLC-Q-TOF-MS/MS was applied to obtain abundant chemical information of metabolites and then the established database, CFM-ID and other software were used to identify and confirm the structures of metabolites. As a result, 20 metabolites of paeoniflorin were found and six metabolites (including four new metabolites) were preliminarily identified. Our strategy had powerful advantages such as good reproducibility, low time consumption, and convenience in operation by imitating the characteristics *in vivo*. Our study was more significant for a better understanding of the metabolic mechanism of paeoniflorin and the complete metabolism process of paeoniflorin *in vivo*.

## Data accessibility

The data supporting the results in this article can be accessed at Dryad Digital Repository: https://doi.org/10.5061/dryad.jb4mq7s [27].
